# Factor structure and short version of the modified Fresno test to assess the use of the evidence-based practice in physiotherapists

**DOI:** 10.1186/s12909-021-02535-9

**Published:** 2021-02-27

**Authors:** Anderson Martins da Silva, Rosimeire Simprini Padula

**Affiliations:** 1 Universidade Cidade de São Paulo, Rua Cesário Galeno 475, São Paulo, SP 03071-000 Brazil; 2 Course of Physical Therapy of Centro Universitario do Vale do Ribeira, UNIVR/UNISEPE, Registro, São Paulo, Brazil; 3 Department of Physical Therapy, Universidade Cidade de São Paulo, São Paulo, Brazil

**Keywords:** Fresno test, Factor analysis, Evidence-based practice, Physiotherapy

## Abstract

**Background:**

The Modified Fresno Test has been used to evaluate the use of the Evidence-Based Physiotherapy (EBP). So far, none of the versions of the Fresno Test were subjected to analysis of the factorial structure. The objective of the study was to describe the exploratory and confirmatory factor structure of the *Modified Fresno Test* adapted to the Portuguese-Brazilian and analyze the statistical feasibility for the elaboration of a short version.

**Methods:**

The questionnaire was applied with a convenience sample of 57 physiotherapists, being 36 professionals (13 of these also professors) and 21 students from the last semester of the physiotherapy course. Exploratory Factor Analysis (EFA) was performed by the method of principal components. Confirmatory Factor Analysis (CFA) was performed by the method of maximum likelihood. The total score of the answers in the test and retest was evaluated, totalling 228 observations. Reliability was assessed by means of internal consistency, using Cronbach’s alpha coefficient.

**Results:**

Reliability was satisfactory (α 0.81) for all questions of the instrument. The coefficient α calculated for the corrected item-total showed values higher than 0.20 except for item 9. Preliminary tests for Exploratory Factor Analysis showed acceptable values with Kaiser-Meyer-Olkin (KMO = 0.80) and Bartlett’s test of sphericity [chi-square (78) = 1149.615, *p* < 0.001], indicating that the correlations were sufficient for analysis. The analysis revealed the presence of 3 factors (eigenvalues> 1), which explains 60.9% of the instrument’s total variance. In Confirmatory Factor Analysis, none of the indices came close to an acceptable level (≥ 0.90), however, the second model which tested a three-factor structure provided a better fit to the data. From the results of this study the Modified Fresno Test short version was drawn.

**Conclusion:**

The analysis showed good factor validity and adequate internal consistency for the use of the instrument consisting of 13 questions and 3 factors. This model proved to be better than the original model. The short version consisting of 9 questions may be an appropriate alternative for use in the population of interest.

**Supplementary Information:**

The online version contains supplementary material available at 10.1186/s12909-021-02535-9.

## Background

Evidence-Based Physiotherapy (EBP) consists of using the best available evidence to guide therapeutic decisions [1–3]. The decision-making process must consider judiciously three requirements [4–7], clinical research of high quality, professional knowledge, and the patient’s preferences. The physiotherapists should follow five steps to effectively translate the evidence into practice: [2–8] 1) convert the need for information in clinical question (s); 2) find the best evidence to answer issues (s); 3) critically assess the validity of the evidence located; 4) fit the evidence into practice considering the professional knowledge and the patient’s preferences, and 5) to evaluate the effectiveness in the execution of the 4 previous stages. The adoption of evidence in practice has been increasingly used by physiotherapists [9, 10]. And generally, the assessment of its effectiveness is conducted by means of self-reported instruments [11].

The *Modified Fresno Test* [12] the adapted version of the Fresno test was [13], developed to evaluate the use of the EBP by physiotherapists. It has been recognized as a reliable instrument for assessing all five steps of EBP objectively [14, 15]. It was adapted for other languages [8–17] and different health professionals [12–20]. In general the Fresno test presents satisfactory measurement properties, however, the responsiveness and structure validity were tested unsatisfactorily [8–16].

The structure validity or items is the capacity that the instrument must measure what it is proposing and can be tested by means of factor analysis [21]. In addition to the assessment of the structure validity, the factor analysis can determine properly the factor structure of the items contained in an instrument, and the contribution and relationship among them [22, 23] Also, it allows to consider the reduction in the number of items of instrument [24, 25]. Furthermore, the analysis of the factor structure of the *Modified Fresno Test* can provide evidence about the power of each structure of the instrument, which was not performed in the original version of the instrument [12]. These analyses will contribute to the definition of the best model for a short version of the instrument. The objective of the study is to describe the exploratory and confirmatory factor structure of the *Modified Fresno Test* adapted to the Portuguese-Brazilian and analyze the statistical feasibility for the elaboration of a short version of the instrument.

## Methods

### Study design

This is a cross-sectional study conducted with a convenience sample of 57 participants. The profile of the participants was based on the characteristics of the instrument and inclusion criteria defined in the study of the development of the instrument [8]. Ethics approval for this study was granted by the Human Research Ethics Committee of the University of Cidade de São Paulo (protocol n° 13,696,713/2012). The inclusion criteria were: (1) professors from Higher Education Institutions public and private institutions inserted in clinical practice, (2) academics enrolled in the last year of the course (3) physiotherapists, regardless of their familiarity with the topic.

### Instrument

The *Modified Fresno Test* [12] for physiotherapists is a self-explanatory instrument that presents an initial text with instructions for completing and two clinical scenarios. The Brazilian-Portuguese version of the instrument was used in this study [8].

The participant must choose one of the scenarios so that, from it, he or she can answer the 13 open-ended questions. The answers to questions 9 and 10 require mathematical calculations. The total score of the instrument was calculated for two independent evaluators by means of the partial score for each question based on qualitative responses from the participants, whose scoring criteria vary from item “a” to the item “d”. The response of each item in question is scored in five categories of classification, namely: (1) non-evident; (2) limited; (3) minimum; (4) strong and (5) excellent (12). The sum of scores of each criterion results in a score per question that varies between 0 and 24 points. The total test score is the sum of points of all questions, which varies between 0 to 224 points [8].

### Procedures

The samplings were carried out in three institutions of Higher Education, in the period from April to September 2013. Professors and academics were recruited from the contact list provided by the responsible department coordinator. Physiotherapists not affiliated with higher education institutions, were contacted by email list and social media. The sample size was estimated as proposed by the guidelines for reliability tests. Each participant received the questionnaire in two moments (test and retest), with an interval of 7 days, allowing the evaluators to score 114 questionnaires. Instructions on the use of the instrument and informed consent form were sent to the participants. After choosing one of the clinical scenarios, the participants should answer the entire test at once with a maximum time of 60 (sixty) minutes. To answer the questionnaire, it was necessary to use a notebook and a calculator. But they were not allowed to use additional features such as *internet websites*, books, etc. All participants answered the questionnaire individually. Out of the total sample, 37 answered the questionnaire in the printed version and 20 on the digital version. There was no difference between groups regarding the administration of the instrument.

### Data analysis

The analysis of the responses and sum of scores of the *Modified Fresno Test* was performed by two independent evaluators with experience in EBP who received a single training, divided into 3 stages of one hour each. The first time was devoted to the guidance on the criteria to score the questions contained in the instrument, the second time to conduct a pilot test, where each evaluator scored 1 test of the sample, and the third time for analysis and discussion of the results of the score in the pilot test.

### Statistical analysis

The structure validity, Exploratory Factor Analysis (EFA) was performed by the method of principal components followed by Confirmatory Factor Analysis (CFA). The total of the 114 questionnaires in the test and retest were analyzed and scored by two trained physiotherapists, totaling 228 responses used to analysis. To investigate the factorability of the instrument the Kaiser-Meyer-Olkin test - KMO and the test of sphericity of Bartlett were used [26, 27]. The KMO index, or adequacy index indicates whether the application of factor analysis is appropriate for the data set [27]. the values between 0.5 and 1.0 indicate that the factor analysis is appropriate [25–29]. Bartlett’s test of sphericity evaluates the null hypothesis of the correlation matrix as matrix-identity [26]. It also evaluates the general significance of all correlations in a matrix of data [24]. Values with significance levels *p* < 0.05 indicate that the matrix is favorable [30]. To determine the number of components to be removed, the criterion of Kaiser-Guttman was used (Eigenvalue > 1) [31]. The procedure of rotation was orthogonal Varimax type [23], as well as the graphic of sedimentation of *“scree plot”.* The factor loadings were considered significant when values were greater than 0.30 [32]. The commonalities were also examined in order to assess the variation of each item [30]. The items that did not have a minimum commonality of 0.4 with the extracted factors, should be considered invalid [25].

**Fig. 1:**
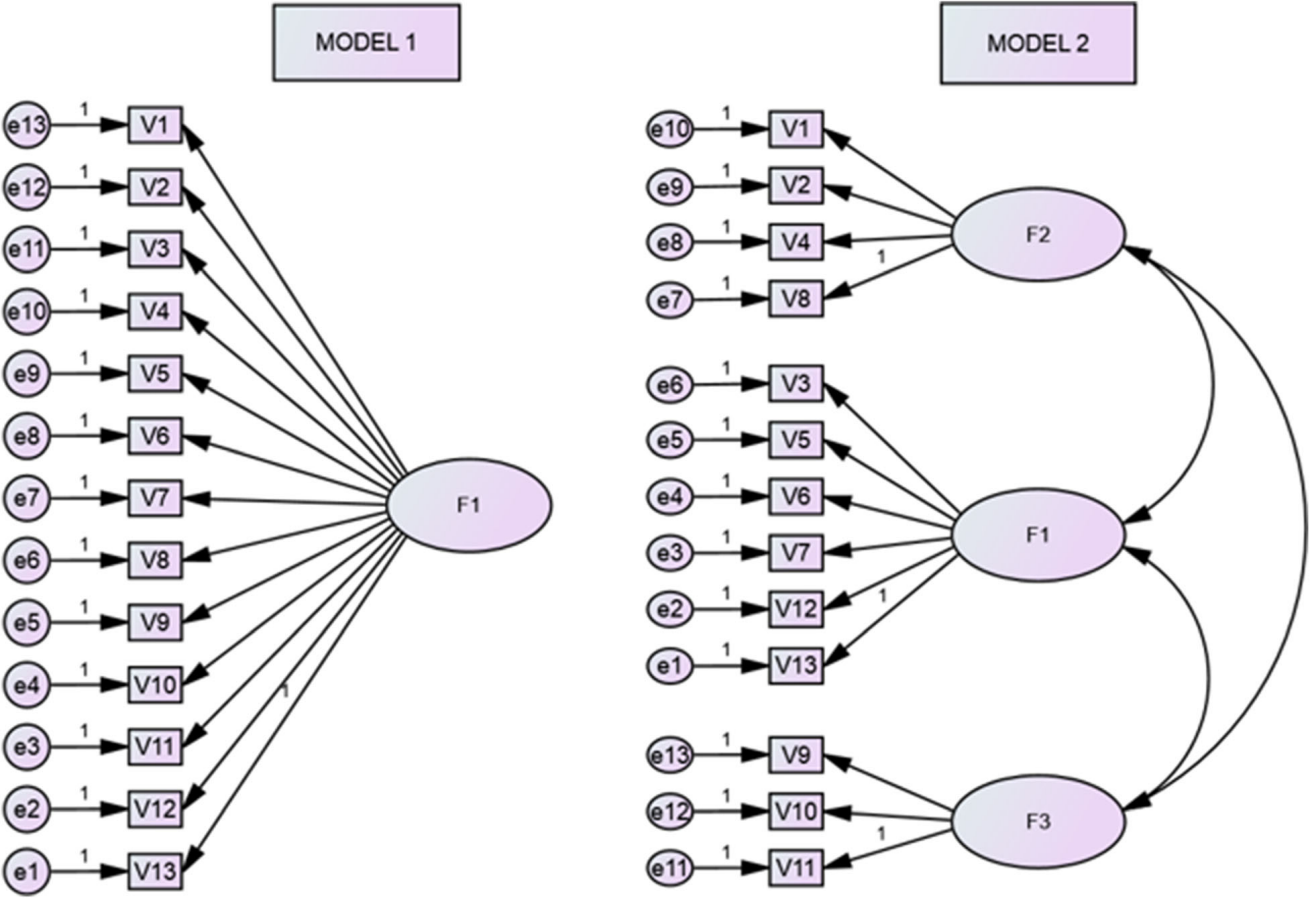
Two models for the confirmatory factor analysis. Model 1 = A general factor. Model 2 = Three factors

Subsequently, it was calculated the CFA to investigate adequacy of EFA model proposed in this study, with the original model defined by Tilson (2010). The model 1 assessed the structure of only a factor of the *Modified Fresno Test* (Fig. 1). The model 2 tested the hypothesis that the instrument is composed of three factors, loaded the 4 items in one latent variable, 6 items for a second variable and 3 items of the instrument for a third latent variable. For the analysis, the maximum likelihood method was used [33, 34]. For adjustment of models the following indexes were used: Index of chi-square (c2), which are estimated values with significance levels (*p* < 0.05), *Goodness of Fit Index* (GFI), *Comparative Fit Index* (CFI), the *Normed Fit Index (NFI)* and the *Non-Normed Fit Index (NNFI)*. Values above 0.90 for these indices indicate a proper fit of the model [35, 36]. The *Root Mean Square Error of Approximation* (RMSEA) considers the error of approximation in the population in a covariance matrix. Values equal to or lower than 0.08 represent a reasonable error [37]. The *Expected Cross-Validation Index* (ECVI) was also analyzed, which indicates the best adjustment of models and it is appropriate to compare non-grouped models [37]. There are no reference values that allow to classify the adjustment of the model, being preferable that it is as low as possible [33–36].

The reliability was evaluated through the analysis of internal consistency, using Cronbach’s alpha coefficient for all items of the instrument and for the corrected item-total correlations [38]. A value of α ≥ 0.7 was defined as “acceptable” and an α ≥ 0.80 as “good”. A value of 0.2 was considered for the corrected item-total correlations [39]. For the data statistical analysis the software *Statistical Package for Social Sciences* (SPSS version 22) and the software *Analysis of Moment Structures* (AMOS - Version 25) were used.

## Results

### Descriptive analysis and reliability

The study carried out with 57 physiotherapists, 36 of them were professionals (13 of these are professors as well) with an average time of formation/performance of 6.6 years (SD-3.8) and 21 students (all of them from the last semester of the course). The internal consistency obtained with Cronbach’s alpha indicated value of 0.81 for the 13 items. The coefficient α calculated for the corrected item-total showed values higher than 0.20 except for item 9. The items 2, 8, 9, 10, 11 and 12 showed the α coefficient lower than 0.50 (Table 1). The mean of scores for individual items ranged from 12.59 (item 1) to 0.57 (item 11).

**Table 1:** Adapted Fresno test mean scores (M) and standard deviation (SD) for individual items, corrected item-total correlation and internal consistency (Cronbach’s alpha) if the item is deleted

Item	M	S.D.	Corrected item-total correlation	Cronbach’s alpha if item is deleted
Q1 – Formulate a clinical question	12.59	7.16	.53	.79
Q2 – Information Sources	10.60	5.98	.42	.80
Q3 – Study design	10.99	7.77	.62	.78
Q4 – Search (search strategy)	11.40	6.40	.62	.78
Q5 – Relevance	7.84	5.55	.62	.79
Q6 – Internal Validity	10.01	7.78	.52	.80
Q7 – Magnitude and significance	8.06	7.03	.63	.78
Q8 – Questioning the patient / family	6.17	4.59	.43	.80
Q9 – Sensitivity, positive predictive value and positive likelihood	1.84	3.60	.10	.82
Q10 – Absolute risk reduction, relative risk, NNT, and *p*-value	1.73	3.90	.23	.81
Q11 – Confidence Interval	.57	1.40	.41	.81
Q12 – Best study design (diagnosis)	1.74	1.99	.46	.81
Q13 – Best study design (prognosis)	1.94	2.00	.54	.81

### Exploratory factor analysis

**Fig. 2:**
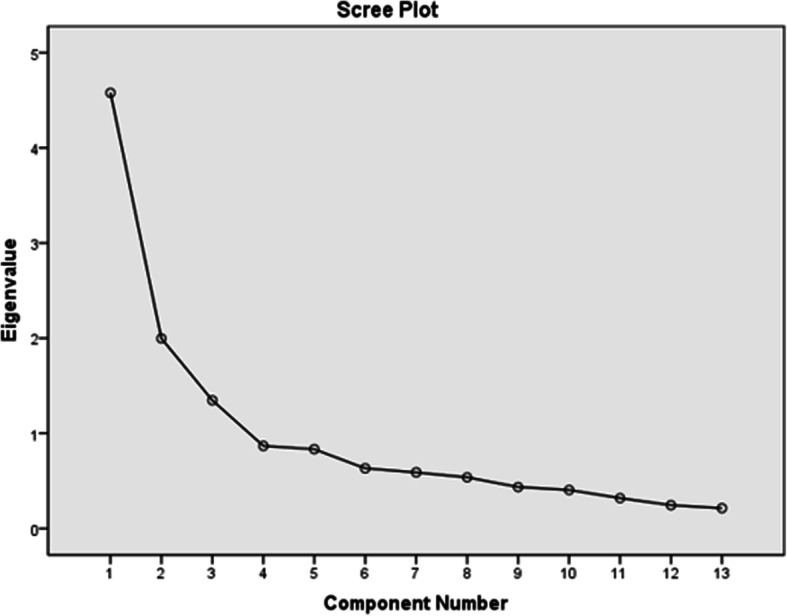
The sedimentation plot (scree plot)

The Kaiser-Meyer-Olkin test verified the suitability of the sample for analysis with acceptable values (KMO = 0.80). The Bartlett’s sphericity test [Chi-square (78) = 1149.615, *p* < 0.001], indicated that the correlations among the items of the instrument are sufficient for the completion of the analysis. The criterion of extraction of factors with eigenvalues, showed the presence of three [3] factors with eigenvalues > 1 related to the 13 items of the instrument, which explains 60.94% of total variance of the participants’ responses (Table 2). These values were satisfactory, as they should explain at least 50% of the total variance of the instrument. The graph of scree plot sedimentation below presents the distribution of the eigenvalues and the three components that are positioned before the inflection point (Fig. 2).

**Table 2:** Total variance explained by 3 (three) components

	Initial Eigenvalues			Extraction Sums of Squared Loadings			Rotation Sums of Squared Loadings		
Factor	Total	% of Variance	Cumulative %	Total	% of Variance	Cumulative %	Total	% of Variance	Cumulative %
1	4.57	35.22	35.22	4.57	35.22	35.22	3.37	25.98	25.98
2	1.99	15.36	50.58	1.99	15.36	50.58	2.59	19.92	45.90
3	1.34	10.36	60.94	1.34	10.36	60.94	1.95	15.03	60.94

Factor 1 grouped the items 3, 5, 6, 7, 12 and 13 that compose the *Modified Fresno Test*. These items had significant loadings with values of α between .50 and .80. Factor 2 grouped the items 1, 2, 4 and 8 with values of factor load between .60 and .70 and factor 3 grouped the items 9, 10 and 11, with values between .50 and .90. Items 3, 4, 5, and 7 were grouped into more than one factor and kept in a certain factor according to their highest factor load. Item 4 was kept in factor 2. Items 3, 4, 5 and 7 were kept in factor 1. Table 3 shows the grouping in each factor of the items of the *Modified Fresno Test* by following the steps in the adoption of EBP. The commonalities per item of the instrument can also be observed in Table 3.

**Table 3:** Principal axis factoring analysis factor loading and communalities (h^**2**^) of the 13 Adapted Fresno test items following varimax rotation

Step/Action	Question - Q	Factor 1	Factor 2	Factor 3	Communalities (h^**2**^)
**Step 1:** Elaboration of the matter	Q1 - Formulate a clinical question		.73		.57
**Step 2:** Search the best available evidence	Q2 - Information Sources		.71		.52
	Q4 - Search (search strategy)		.62		.57
**Step 3a:** Critical evaluation (qualitative) evidence	Q3 - Study design	.66			.59
	Q5 - Relevance	.52			.54
	Q6 - Internal Validity	.71			.54
	Q7 - Magnitude and significance	.60			.55
	Q12 - Best study design (diagnosis)	.83			.69
	Q13 - Best study design (prognosis)	.88			.77
**Step 3b:** Critical evaluation (quantitative) evidence	Q9 - Sensitivity. positive predictive value and positive likelihood			.90	.83
	Q10 - Absolute risk reduction. Relative risk. NNT. and p-value			.91	.84
	Q11 - Confidence Interval			.51	.42
**Step 4:** Implementation of evidence in clinical practice	Q8 - Questioning the patient / family		.69		.49

### Confirmatory factor analysis

The model 1, which tested the structure of a factor of the *Modified Fresno Test* did not provide an adequate adjustment (Table 4). None of the indices approached an acceptable level (≥ 0.90). The model 2 which tested a three-factor structure provided a better fit to the data. Although the chi-square test was significant, the difference of the chi-square test between the model 1 and 2, was statistically different (χχ2 = 237.56, df = 62; *p* < 0.001). Therefore, there was a better adjustment of the model 2 in comparison with the model 1. However, the indices of adjustment of the model 2 also did not reach the acceptable level. Thus, the model 2, although better than the model 1 also did not provide a proper fit to the data.

**Table 4:** Fit indices for the three PSWQ factor models tested according to confirmatory factor analysis

Models	***X***^***2***^	df	GFI	CFI	RMSEA	SRMR	AIC	ECVI	NFI	NNFI
Model 1	492.14	65	.75	.61	.17	.12	544.14	2.40	.58	.50
Model 2	254.58	62	.85	.82	.11	.10	312.58	1.38	.78	.73

*CFI* Comparative Fit Index, *GFI* Goodness of Fit Index, *RMSEA* Root Mean Square Error of Approximation, *SRMR* Standardized Root Mean Residual (SRMR), *AIC* Akaike’s Information Criterion, *ECVI* Expected Cross-Validation Index, *NFI* Normed Fit Index; and *NNFI* Non-Normed Fit Index

### Elaboration of the short version of the *Modified Fresno Test*

From the results of this study the *Modified Fresno Test* short version was drawn. The short version of the instrument consisted of the exclusion of 4 items, based on the arguments set out by the authors of this study. The arguments for the exclusion of items were the values obtained by the Cronbach’s alpha coefficient for reliability and the values of the factor structure. To contribute to the decision-making process, a group of experts was constituted (*n* = 16) composed by postgraduate students and professionals with training and knowledge about evidence-based practice. Previously, it was sent to the members of the group of experts the article of Brazilian-Portuguese version of *Modified Fresno Test* for Physiotherapists [7]. Then, it was held two face-to-face meetings (3 h long) to discuss the items of the instrument and the test results of measurement properties. From the debate, the conclusions allowed to consider the exclusion of items 2, 9, 10 and 11. Thus, the short version contains 9 items and contemplates the items 1, 3, 4, 5, 6, 7, 8, 12 and 13 (Additional files 1 and 2). After the exclusion of these four items, the internal consistency indicated value (α = 0.82) for all the items of the instrument.

## Discussion

The Exploratory Factor Analysis demonstrated suitability of the *Modified Fresno Test*. It showed good internal consistency, with the values of α satisfactory for all the extracted factors. This property of measurement has sustained the reliability of the instrument in its most varied versions. Three extracted factors with eigenvalues > 1, shows a small total variance of participants’ responses and provides greater reliability of the instrument. Items 9 and 10 showed the highest loadings among the 13 items analyzed, which demonstrates the important items for the structure of instrument. However, they showed low values by Cronbach’s alpha reliability. They are items that require a high knowledge of the participants on statistics. The confirmatory factor analysis tested the proposed model with 3 factors (model 2) in comparison to the original version of the instrument. 9 indices were analyzed for adjustments using the method of maximum likelihood. The indices that represent the proportional improvement in the adjustment of models (CFI, NFI and NNFI), showed a better adjustment of the model 2. However, the analysis showed that none of the tested models showed appropriate adjustments.

The internal consistency by Cronbach’s alpha coefficient showed satisfactory reliability for all items of the instrument. These values agree with reliability values presented in *original Fresno Test studies* developed by Ramos et al. (2003), Argimon-Pall’as et al. (2010), Tilson (2010) and Silva et al. (2015) [8–17]. These studies claim that the test must be reliable to different languages and professionals. In the analysis by item of the instrument, 2 items (9 and 10) values were not acceptable. Items 9 and 10 assess the participants’ knowledge in performing statistical calculations instead of interpreting the statistical results for clinical decision-making. This makes these items unable to respond what they propose. Item 11 evaluates the interpretation of the confidence interval for statistical significance and presented low reliability, in addition to minimum value for commonality. The results demonstrated that these items do not contribute significantly to the overall reliability of the instrument. The experts’ opinion group pointed to a need for an approach of items 9 and 10 related to the interpretation of the results and not to perform statistical calculations. Also, they evidence that the statistical concepts required in these items are already covered in other items of the instrument, as for instance, the item 7. The low reliability of these items justifies its high omission of responses, reported by Silva et al. (2015) [8] and may be related to the difficulty in understanding the questions. The difficulty dealing with statistics is one of the main obstacles, among others pointed to the adoption of EBP in several studies on the theme.

From the results obtained in the exploratory and confirmatory factor analysis, the making-decision was taken to introduce a short version of the *Modified Fresno Test* which consisted of the exclusion of 4 items of the instrument. The version proposed also enables the instrument to evaluate all stages of adoption of EBP objectively. The version consisted of 9 items presented with better reliability in relation to the version composed of 13 items. This short version may be an appropriate alternative to be used in the population of interest. The sample used in this study may characterize a limitation. Just as in other studies of adaptation, a convenience sample was used instead of determining a sample size to achieve statistically significant results. It is worth noting that more research should be conducted in order to confirm the structure of the instrument. In this sense, it is suggested to conduct studies that compare the models presented here with other alternative models. Still, it would be important to attest to the ability of the instrument to discriminate among theoretically different groups. These analyses can further enhance the instrument.

## Conclusion

The *Modified Fresno Test* in Brazilian-Portuguese version demonstrated satisfactory factor validity and good internal consistency. The results of the confirmatory factor analysis showed that the rates of adjustment of the model 2 composed of three factors, proved to be more suitable than the model 1. These results are enabled to assert that the short version presented to the instrument can be a very suitable alternative to be used in the population of interest.

## Supplementary Information

**Additional file 1.**

**Additional file 2.**

## Data Availability

The datasets used and/or analyzed during the current study are available from the corresponding author on reasonable request.

## Abbreviations

EBP: Evidence-Based Physiotherapy
EFA: Exploratory Factor Analysis
CFA: Confirmatory Factor Analysis
KMO: Kaiser-Meyer-Olkin test
GFI: Goodness of Fit Index
CFI: Comparative Fit Index
NFI: Normed Fit Index
NNFI: Non-Normed Fit Index
RMSEA: Root Mean Square Error of Approximation
ECVI: Expected Cross-Validation Index
SPSS: Statistical Package for Social Sciences
AMOS: Analysis of Moment Structures
