# Supplementary material for: Factor structure and short version of the modified Fresno test to assess the use of the evidence-based practice in physiotherapists
Source: BMC Med Educ. 2021 Feb 27;21:135. doi: 10.1186/s12909-021-02535-9 (PMC7912886; doi:10.1186/s12909-021-02535-9)
Supplement: Supplementary file 1 — Additional file 1. [file 12909_2021_2535_MOESM1_ESM.doc]

**Apêndice 1- Versão curta do *Teste Modificado de Fresno* para fisioterapeutas**

**Instruções:**

Prática Baseada em Evidências (PBE) envolve conhecimentos e habilidades relacionadas com a identificação e avaliação de evidências para informar a prática. Esta ferramenta, o Teste Modificado de Fresno é projetado para avaliar suas habilidades para a PBE.

Há sete perguntas de resposta curta, e 2 questões de preenchimento de lacunas. Uma calculadora e papel para anotação lhe foram fornecidos. Recursos adicionais (sites da internet, livros, etc.) não são permitidos.

Por favor, preencha todo o teste de uma só vez e permita-se até 60 minutos para completar o teste.

------------------------------------------------------------------------------------------------------------------------------------------------------------------

Responda às perguntas 1 a 3 e 7 com base nos seguintes cenários clínicos:

**Cenário 1**: Você acabou de avaliar Maria, uma secretária, que recentemente sofreu uma lesão lombar relacionada ao trabalho, movimentando caixas de arquivos de 4,54 Kg há 3 dias atrás. Suas radiografias são negativas e seu único sintoma é a dor na coluna lombar com intensidade 2/10, durante movimentos de flexão e ficar sentada por tempo prolongado. Ela ficou fora do trabalho por dois dias e está ansiosa para voltar, mas está também preocupada com uma nova lesão. Você está pensando em um programa de exercícios de estabilização, mas pergunta a si mesmo se a terapia manual deve ser incluída no programa de fisioterapia do paciente.

**Cenário 2**: Marvin é um garoto de 10 anos com hemiparesia secundária, devido a um acidente vascular cerebral associado a uma má formação artério-venosa. Ele apresenta-se à terapia ambulatorial e seus pais expressam preocupação particular sobre o braço de Marvin e fraqueza nas pernas. Você está pensando em implementar um programa intensivo de tarefa específica de fortalecimento, mas um colega adverte que tal programa é capaz de aumentar o tônus flexor e espasticidade moderada do paciente e sugere baixa intensidade de alongamento e um programa de posicionamento passivo.

| **Pergunta # 1:Escolha um dos cenários clínicos acima. Escreva uma pergunta clínica, focada neste cenário, que irá ajudá-lo a organizar uma busca na literatura clínica.** |
| --- |
| Resposta: |

| **Pergunta # 2: Que tipo de estudo (projeto de estudo) melhor responderia a sua pergunta clínica descrita na pergunta 01 e por quê?** |
| --- |
| Resposta: |

| **PERGUNTA # 3: Se você estivesse em uma busca no Medline, CINAHL ou qualquer outro banco de dados para a pesquisa original, para responder a sua pergunta clínica relacionada ao cenário selecionado para a pergunta 1, descreva a estratégia de busca que você pode usar. Seja o mais específico possível sobre os termos de pesquisa e campos de pesquisa que você usaria. Explique sua justificativa para esta abordagem. Descreva como você pode limitar sua pesquisa, se necessário, e explique o seu raciocínio.** |
| --- |
| Resposta: |

| **Pergunta # 4: Quando você encontrar um artigo sobre à sua pergunta clínica ou quaisquer outras, que características do estudo você vai considerar para determinar se ele é relevante? Inclua exemplos. As questões 5 e 6 irão perguntar a você como determinar se o estudo é válido, e quão importante são os resultados. Para esta pergunta, por favor, concentre-se em como determinar se ele é realmente relevante para a sua prática.** |
| --- |
| Resposta: |

| **PERGUNTA # 5: Quando você encontrar um artigo relacionado à sua pergunta clínica ou quaisquer outras, que características do estudo você vai considerar para determinar se as suas conclusões são válidas? (Você já foi abordado sobre relevância e a questão 6 vai perguntar como determinar a importância dos resultados. Para esta pergunta, por favor, concentre-se na validade do estudo).** |
| --- |
| Resposta: |

| **Pergunta # 6: Quando você encontrar um artigo, que se relacione com a sua questão clínica ou quaisquer outras, que características dos resultados você vai considerar para determinar sua magnitude e significância (clínica e estatística)?** |
| --- |
| Resposta: |

| **Pergunta # 7: Para o cenário clínico que você escolheu, liste duas questões que você gostaria de fazer ao paciente / família para obter uma melhor compreensão de suas preferências pessoais e / ou circunstâncias em relação a sua questão clínica.** |
| --- |
| Resposta: |

| **Pergunta # 8: Qual desenho do estudo é o melhor para um estudo sobre o diagnóstico?** |
| --- |
| Resposta: |

| **Pergunta # 9: Qual desenho do estudo é o melhor para um estudo sobre o prognóstico?** |
| --- |
| Resposta: |

**Apêndice 2- Escore de pontuação da versão curta do *Teste Modificado de Fresno*** para fisioterapeutas

| **Pergunta # 1: Escolha um dos cenários clínicos acima. Escreva uma pergunta clínica, focada neste cenário, que irá ajudá-lo a organizar uma busca na literatura clínica.** | | | | |
| --- | --- | --- | --- | --- |
|  | **Excelente**  6 pontos | **Forte**  4 pontos | **Limitado**  2 pontos | **Não evidente**  0 pontos |
| a: População | Vários descritores relevantes; por exemplo, “lesão relacionada com o trabalho”, “mulher” ou “grave” ou “dor lombar”, por exemplo, "menino com hemiparesia" grupo etário específico, sexo, diagnóstico, apresentação motora. | Um descritor apropriado como os exemplos acima, "Mulheres" ou "trabalhador" ou "dor lombar", por exemplo, "hemiparesia" menino "" "10 anos" "pós-AVC" | Um único descritor geral capaz de contribuir para pesquisa; por exemplo, "Paciente" | 0 pontos  Nenhum dos presentes acima. |
| b: Intervenção | Inclui intervenção de interesse específico; (intervenção poderia ser uma técnica de diagnóstico);  • terapia manual;  • componentes específicos individuais de terapia manual;  • combinação de exercício e terapia manual;  • tarefas específicas de fortalecimento. | Não se aplica | Menciona intervenção, mas é capaz de contribuir para pesquisa; por exemplo, "Métodos" "opções" "tratamentos”. | Nenhum dos presentes acima. |
| c: Comparação | Identifica alternativa de interesse específico; exemplo "Nenhuma terapia manual", "alongamento de baixa intensidade". | Não se aplica | Menciona comparação, mas é capaz de contribuir para pesquisa; exemplo “Métodos alternativos" | Nenhum dos presentes acima. |
| d:Resultado | Resultado que é objetivo e significativo para o paciente ou o caso do paciente (se a pergunta é de diagnóstico, deve estar relacionado com o que o diagnóstico está tentando detectar); exemplo, retorno ao trabalho, redução da dor, prevenção de lesões; por exemplo, controle motor seletivo ou uso funcional de extremidades paralisadas, velocidade no andar. | Sem resultado específico  • Recuperação  • Espasticidade  • Tônus  • Força | Referência ao resultado, mas de modo geral a ser capaz de contribuir para pesquisar:  • Efeitos  • Alteração do resultado  • Eficaz  • Melhoria  • Sucesso | Nenhum dos presentes acima. |

| **Pergunta # 2: Que tipo de estudo (projeto de estudo) melhor responderia a sua pergunta clínica descrita na pergunta 01 e por quê?** | | | |
| --- | --- | --- | --- |
|  | **Excelente**  12 pontos | **Forte**  9 pontos | **Limitado**  6 pontos |
| a:Projeto de estudo | Nomeia uma das melhores fontes:  • Experiência Controlada Aleatorizada;  • Experiência Aleatorizada;  • Revisão Sistemática;  • Meta-Análise;  • Experiência Aleatorizada, duplamente cego. | Descreve, mas não chama pelo nome, uma das melhores fontes como acima:  • Comparando dois grupos, um obtém-se o tratamento, o outro placebo;  • Estudo duplamente cego. | Descreve ou nomeia um projeto de estudo menos desejável:  • Estudo de coorte;  • Ensaio clínico prospectivo;  • Meta-análise de tais estudos;  • Longitudinal ou prospectivo. |
| b: Justificativa | Inclui justificativa bem fundamentada, que reflete a compreensão da importância da aleatorização e / ou cegueira. Conecta explicitamente aleatorização para redução de confusão e / ou cegueira de observador ou viés de aferição. Por exemplo: "Um estudo controlado aleatorizado tentará evitar qualquer viés que possa influenciar o resultado do estudo através de aleatorização" OU "mais adequada para questões de terapia, pois reduz preconceitos e controles para fatores de confusão." | A justificativa está presente, e toca em questões relacionadas com a aleatorização e / ou cegueira, mas menos claramente articulada, por exemplo, "Os grupos devem ser semelhantes" ou "tentar eliminar fatores de confusão" ou "evitar viés de seleção" ou "ser objetivo" ou "eliminar o preconceito". | A justificativa está presente, e levanta questões legítimas não relacionados à aleatorização ou cegueira, tal como a relação custo-benefício, recolhimento de produto.  Talvez mencionar aleatorização ou cegueira, mas sem explicação. (Por exemplo: "o melhor em um cenário aleatório e cego"),  Por exemplo: "Opiniões de gráfico fornecem muitos dados sem muito custo" |

| **PERGUNTA # 3: Se você estivesse em uma busca no Medline, CINAHL ou qualquer outro banco de dados para a pesquisa original, para responder a sua pergunta clínica relacionada ao cenário selecionado para a pergunta 1, descreva a estratégia de busca que você pode usar. Seja o mais específico possível sobre os termos de pesquisa e campos de pesquisa que você usaria. Explique sua justificativa para esta abordagem. Descreva como você pode limitar sua pesquisa, se necessário, e explique o seu raciocínio.** | | | | |
| --- | --- | --- | --- | --- |
|  | **Excelente**  8 pontos | **Forte**  6 pontos | **Limitado**  3 pontos | **Não evidente**  0 pontos |
| a:Termos de busca | 3 ou mais termos que refletem paciente, intervenção, comparação, e resultado (PICR) sendo considerados. | 2 termos do PICR. | 1 termo do PICR. | Não presente |
| b:  Instruções/ Estratégia | Descrição da estratégia de pesquisa reflete o entendimento de que os artigos no banco de dados são indexados por mais de um campo. Discute um ou mais campo/índice/rótulo pelo nome (MESH, Palavra do título, nome da publicação, idioma, palavras-chave, autor, título da revista, utilização de operadores booleanos, etc.) **E** fornece justificativa plausível para a estratégia de busca usando um ou mais destes índices, por exemplo, “Palavra-chave é menos específico do que a estrutura". | Nomeia um ou mais campo ou índice de categoria, mas não fornece defesa plausível de estratégia de pesquisa com base neste conhecimento, por exemplo, "Eu faria uma pesquisa por palavra... seguido por..." "Eu usaria termos... desta forma". | Fraca descrição da estratégia, nenhum nome dado aos rótulos, ou estratégia abertamente equivocada  Por exemplo: "Gostaria de usar termos..." [nenhuma descrição da estratégia]. | Nenhuma compreensão evidente para os artigos "marcados" por diversos campos ou índices. |
| c:Delimitadores | Descreve mais de uma abordagem para a busca de limitação (por exemplo, "limite humano" ou "Inglês" ou "adulto"), nomeia um tipo de publicação específica, ou descrição de consultas clínicas no PubMed, ou o uso de operadores booleanos ou pesquisa combinações ou inclui termos relacionados a um projeto de estudo ideal (por exemplo, ao acaso) ou sugere uso de subposições.  * NOTA: Se o assunto inclui o nome do índice ao descrever um delimitador (por exemplo, "verificar a linguagem como Inglês"), então nós damos crédito para um rótulo, assim como a um método de delimitação. | Descreve apenas um método comum de limitar a pesquisa. Por exemplo, descreve as formas de estreitar a pesquisa usando palavras-chave, mas nenhuma outra das estratégias listadas. | Fornece uma explicação fraca ou descrição do uso de limitadores / estreitamento de busca. | Nenhuma técnicas válidas para limitar uma pesquisa listada. |

| **Pergunta # 4: Quando você encontrar um relatório de pesquisa original sobre esta questão, ou quaisquer outras, que características do estudo você vai considerar para determinar se ele é relevante? Inclua exemplos. Questões 5 e 6 irão perguntar a você como determinar se o estudo é válido, e quão importante são os resultados. Para esta pergunta, por favor, concentre-se em como determinar se ele é realmente relevante para a sua prática.** | | | | |
| --- | --- | --- | --- | --- |
|  | **Excelente**  12 pontos | **Forte**  9 pontos | **Limitado**  5 pontos | **Não evidente**  0 pontos |
| a: Questão | Discussão bem-fundamentada e refletida da relevância das variáveis ​​dependentes e independentes utilizadas no estudo, incluindo exemplos / razões específicas. Pode discutir (bem-fundamentada e refletida):  • A viabilidade do teste ou intervenção;  • "O teste pode funcionar, mas se a minha prática não pode se dar ao luxo de comprar a máquina, não importa";  • O paciente ou doença orientada natureza do resultado;  • "Elas medem a capacidade das crianças para usar a função melhorada em atividades lúdicas?"  • A congruência entre a definição operacional e o a questão de pesquisa, por exemplo, "Se o seu método de aferição do resultado é uma representação realista do resultado que nos interessa". | Menos discussão reflexiva da relevância das variáveis ​​dependentes e independentes utilizadas no estudo.  Pode incluir conceitos ou exemplos específicos sem razão clara.  Pode referir-se aos mesmos itens listados na "excelente", mas sem demonstrar profundidade de compreensão. | Resposta implica na consideração de quão bem o estudo aborda a questão em mãos, mas oferece pouca discussão sobre por que isso pode ser importante,  • Por exemplo: "Quais são as variáveis?";  • "Ela respondeu à minha questão?";  • "O desfecho";  • "O propósito do estudo";  • "Impactará minha prática?";  • “Comprimento de acompanhamento". | Nenhuma discussão sobre a questão de pesquisa e variáveis ​​utilizadas para respondê-la. |
| b: Descrição dos assuntos | Inclui ambos:  Uma expressão clara da importância da relação entre os sujeitos do estudo e da população alvo. **E**  pelo menos um exemplo de uma doença ou característica demográfica pertinente.  • Por exemplo: "Os pacientes eram semelhantes ao meu em termos de idade e raça?" ou  • "era um amostra hospitalar ou clínica como os meus pacientes?" ou  • "Os pacientes tinham mesmo nível de gravidade da doença como meu paciente?" ou  • "fez seleção ou inclusão inadequado de critérios dos resultados em uma população que difere da minha em raça, idade, etc.". | Inclui um, mas não ambos:  Uma expressão clara da importância da relação entre os sujeitos do estudo e da população alvo,  **ou**  pelo menos um exemplo de uma doença relevante ou característica demográfica  Por exemplo, "O paciente é como o meu?" ou” o nível de educação da população". | Resposta indica a consideração dos sujeitos do estudo, mas não oferece nenhuma discussão sobre a conexão entre sujeitos do estudo e a população alvo ou características específicas da amostra  • Por exemplo: "É uma amostra adequada?" ou  • "qual foi à resposta ou taxa de participação?" ou  • "quais foram os critérios de exclusão?" ou  • "viés de seleção" ou  • "ajuste" ou  • "onde o estudo foi conduzido". | Nenhuma discussão sobre as características dos sujeitos de pesquisa. |

NOTA: As respostas às perguntas 4, 5 e 6, podem ser aplicadas a qualquer parte da rubrica de classificação para esses itens

| **PERGUNTA # 5: Quando você encontrar um relatório de pesquisa original relacionado à sua pergunta clínica ou quaisquer outras, que características do estudo você vai considerar para determinar se as suas conclusões são válidas? (Você já foi abordado sobre relevância e a questão 6 vai perguntar como determinar a importância dos resultados. Para esta pergunta, por favor, concentre-se na validade do estudo).** | | | | | |
| --- | --- | --- | --- | --- | --- |
|  | **Excelente**  24 pontos | **Forte**  18 pontos | **Limitado**  10 pontos | **Mínimo**  5 pontos | **Não evidente**  0 pontos |
| a: Validade Interna | Lista ou descreve pelo menos 5 questões importantes para a validade interna, tais como:  • Adequação do projeto de estudo;  • Adequação da cegueira;  • Cancelamento de Alocação;  • Aleatorização de trabalho de grupo;  • Medição inválida ou tendenciosas ("seguido próprio protocolo?");  • Importância do grupo de comparação ou controle;  • Intenção de tratar a análise;  • Consideração de co-variáveis ​​apropriadas ("foram outros fatores relevantes considerados?");  • Conclusões consistentes com a evidência ("Os resultados fazem sentido?");  • Importância do acompanhamento de todos os participantes do estudo;  • Análise estatística apropriada;  • Tamanho da amostra /Poder;  • Patrocínio;  • Quando o estudo foi realizado;  • Confirmação com outros estudos;  • Medidas de resultado válidas. | Identifica 3-4 questões específicas como acima. | Identifica duas questões específicas como acima. | Menciona validade interna ou um conceito específico da lista de exemplos acima. | Nenhum dos presentes acima. |

NOTA: As respostas às perguntas 4, 5 e 6, podem ser aplicadas a qualquer parte da rubrica de classificação para esses itens

| **Pergunta # 6: Quando você encontrar um artigo, que se relacione com a sua questão clínica ou quaisquer outras, que características dos resultados você vai considerar para determinar sua magnitude e significância (clínica e estatística)?** |
| --- |

|  | **Excelente**  12 pontos | **Forte**  9 pontos | **Limitado**  5 pontos | **Não relevante**  0 pontos |
| --- | --- | --- | --- | --- |
| a: Magnitude | Resposta deve discutir claramente ambos:  • Significado clínico ("qual é o significado clínico?" Ou "quão ampla foi à diferença encontrada", a alteração excede MCID)  E  • Exemplo (s) de tamanho de efeito de medições (por exemplo, especificidade, sensibilidade, razão de probabilidade de um teste, número necessário para tratar, o risco relativo, redução do risco absoluto, diferença média de resultados contínuos, positivo ou negativo, o valor preditivo). | Resposta discute um, mas não ambos:  • Significado clínico ("qual é o significado clínico?" Ou "quão ampla foi à diferença encontrada?")  OU  • Exemplo (s) tamanho de efeito de medições (por exemplo, especificidade, sensibilidade, razão de probabilidade de um teste, número necessário para tratar, o risco relativo, redução do risco absoluto, diferença média de resultados contínuos, positivo ou negativo, o valor preditivo. | Resposta só sugere uma consideração de significado clínico ou o tamanho do efeito.  • Por exemplo: "Será que isso importa?" "será que vai impactar minha prática" ou  • Por exemplo: (menciona "mínima diferença clinicamente importante", mas não explica como esse valor seria usado para determinar a significância clínica). | Nenhum dos presentes acima. |
| b:  Significância Estatística | Discussão refletida e bem-fundamentada dos índices de significância estatística, incluindo pelo menos dois exemplos específicos de importantes conceitos relacionados, tais como:  • P- valores;  • Intervalos de confiança;  • Poder;  • Precisão das estimativas;  • Erro Tipo 1 ou tipo 2. | Lista mais do que um conceito (como acima) com a discussão insuficiente ou ausente (por exemplo, "p-valor e intervalos de confiança")  OU  Lista e discute apenas um conceito (por exemplo, "p-valor menor que <. 05"). | Menciona a necessidade de avaliar a significância estatística ou nomeia somente um dos conceitos acima sem mais discussão (por exemplo, "p-valores", "estatisticamente significativos"). | Nenhum dos presentes acima |

NOTA: As respostas às perguntas 4, 5 e 6, podem ser aplicadas a qualquer parte da rubrica de classificação para esses itens

| **PERGUNTA # 7: Para o cenário clínico que você escolheu, liste duas questões que você gostaria de fazer ao paciente / família para obter uma melhor compreensão de suas preferências pessoais e / ou circunstâncias em relação a sua questão clínica.** | | | | |
| --- | --- | --- | --- | --- |
|  | **Excelente**  8 pontos | **Limitado**  4 pontos | **Mínimo**  2 pontos | **Não evidente**  0 pontos |
| a: Questão1 | A pergunta é apropriada para obter informações importantes sobre as preferências do paciente, os valores, as circunstâncias, as expectativas, e / ou motivações que irão impactar diretamente os cuidados clínicos. | A pergunta é geral, mas trata de questões relevantes para a compreensão da perspectiva do paciente. | A pergunta é geral e não trata de questões específicas para as perspectivas do paciente.  Por exemplo: Pergunta padrão de avaliação subjetiva não específica para perspectivas do paciente.  Por exemplo: Perguntas sim / não ou factual que não são susceptíveis de trazer à tona detalhes sobre perspectiva do paciente. | Nenhuma pergunta ou não uma questão atual  • "histórico médico antigo" ou  • "preferências" |
| b: Questão2 | 8: Mesmo que acima, mas traz à tona informações diferentes do que a primeira pergunta (caso contrário 0). | 4: Mesmo que acima, mas traz à tona informações diferentes do que a primeira pergunta (caso contrário 0). | 2: O mesmo que acima, mas traz à tona informações diferentes do que a primeira pergunta (caso contrário 0). | 0: Nenhuma pergunta ou não uma questão real. |

Arredondamento é aceitável (por exemplo 21,9-22 é aceitável)

| **Pergunta # 08: Qual desenho do estudo é o melhor para um estudo sobre o diagnóstico?** | | |
| --- | --- | --- |
|  | **Excelente**  4 pontos | **Não evidente**  0 pontos |
| A (resposta) | Estudo de Coorte; estudo transversal; comparação com o padrão ouro; revisão sistemática. | Outros |

| **Pergunta # 9: Qual desenho do estudo é o melhor para um estudo sobre o prognóstico?** | | |
| --- | --- | --- |
|  | **Excelente**  4 pontos | **Não evidente**  0 pontos |
| A (resposta) | Coorte; prospectivo, longitudinal; Revisão Sistemática. | Outros |
